# Associations between Food Insecurity and Depression among Diverse Asian Americans

**DOI:** 10.31372/20200504.1114

**Published:** 2021

**Authors:** Sonia Lai, Deborah Huang, Indraneil Bardhan, Mijung Park

**Affiliations:** a University of California, San Francisco, United States; b University of Washington, United States

**Keywords:** food insecurity, depression, Asian Americans, gender differences, immigrants

## Abstract

**Background:** Proper nutrition is an essential component to both physical and emotional health. Food insecurity (FI) is a potentially critical public health problem. The link between FI and elevated risk for depression has been well documented. Yet, it is largely unknown how diverse older adult populations experience FI differently. Therefore, the aims of this study were to examine how gender, race/ethnicity, and nativity may impact the magnitude of the association between FI and depression.

**Methods:** We used a nationally representative sample of the Asian American population from the National Latino and Asian American Study (NLAAS). We built logistic regression models with major depression in the past 12 months as the dependent variable, and FI as the independent variable. Several demographic and socioeconomic characteristics were added to the models to control for potential biases. All statistical estimates were weighted, using the recommended NLAAS sampling weight, to ensure representativeness of the US population.

**Results:** About 35% (weighted adjusted 95% CI: 29.49–39.00) of Asian Americans experienced some level of FI at the time of survey. Experiencing FI over the past 12 months increased the likelihood of having clinical depression (weighted adjusted odds ratio: 1.44, weight adjusted confidence interval: 0.79–2.10). The magnitude of associations between FI and depression varied by race/ethnicity (*F* (7, 47) = 6.53, *p* (3, 41) = 10.56, *p* (3, 41) = 9.85).

**Conclusions:** Food insecurity significantly increases the likelihood of clinical depression among Asian Americans. Greater attention is needed towards food-insecure Asian Americans and their mental health.

## Introduction

Food insecurity (FI hereafter) is defined as a household-level economic and social condition of limited or uncertain access to adequate food (United States Department of Agriculture, n.d.-a), and affects about one in nine Americans (Coleman-Jensen, Rabbitt, Gregory, & Singh, 2019). In 2019, about 13.7 million (10.5%) US households were food insecure at some time, making food insecurity an urgent national public health concern (United States Department of Agriculture, n.d.-b)

Although FI can impact all individuals, literature has shown that it disproportionately affects minority, immigrant, and low-income populations (Myers & Painter, 2017). Unfortunately, existing data on FI in the United States do not include detailed information about Asian Americans and limited knowledge is available on how FI impacts Asian American communities. One of the few available publications on the topic is from California (Becerra, Mshigeni, & Becerra, 2018). In California, the highest prevalence of food insecurity was found among Vietnamese individuals (16.42%) and the lowest prevalence was among Japanese individuals (2.28%). The same study also noted that significant differences in rates of FI were attributed to low acculturation and immigrant status among Chinese, Korean, and Vietnamese subgroups. Despite these findings, there is a clear gap in data regarding the discussed demographics. This lack of information is concerning, as Asian Americans are one of the fastest growing segments of the US populations.

Studies have shown a strong link between FI and depression (Arenas, Thomas, Wang, & DeLisser, 2019). Several segments of populations experiencing FI have been identified to have elevated risk for depression. For example, compared to their food-secure counterparts, older adults with FI are at higher risk of depression, and exacerbation of depressive symptoms (Ziliak & Gundersen, 2014). Medically vulnerable older adults are also at higher risk of developing depression when they experience FI (Montgomery, Lu, Ratliff, & Mezuk, 2017).

Despite the strong evidence regarding links between FI and depression, insufficient data is available to inform us about how FI may be associated with concurrent depression among Asian Americans. Furthermore, few studies have examined how FI impacts diverse Asian American subgroups. Additionally, existing studies identified depression using research tools measuring depressive symptom severity, which are inadequate for the formal diagnosing of clinical depression. Our goals are to address these knowledge gaps by estimating the association between FI and clinical depression over the past 12-month period, and by assessing how said link may vary by gender and by race/ethnicity.

## Methods

### Study Sample

This is a cross-sectional, descriptive study, using data from the National Latino and Asian American Study (NLAAS), a nationally representative epidemiological study of mental health among Asian and Latino populations (Alegria et al., 2004; Pennell et al., 2004). The sampling design has been well documented elsewhere (Heeringa et al., 2004; Pennell et al., 2004). The selection of a probability sample of respondents required a four-step sampling process: a primary stage sampling of US Metropolitan Statistical Areas and counties, a second stage sampling of area segments, a third stage sampling of housing units within the selected area segments, and a fourth stage sampling of the random selection of eligible respondents from the sample housing units. The weighted response rates for the combined NLAAS samples of primary and second adult respondents were 73.2% for the total sample, 75.5% for the Latino sample, and 65.6% for the Asian sample.

Data collection took place between May 2002 and November 2003. Eligibility criteria to be included in the study were: 18 years of age or older, reside in noninstitutional settings in one of 50 states of the United States or District of Columbia, and identify self as of Latino, Hispanic, or Spanish descent, or of Asian descent. The NLAAS instrument was administered in the respondent’s choice of languages (English, Spanish, Chinese, Vietnamese, or Tagalog) by fully bilingual lay interviewers. Interviews were conducted face-to-face unless respondents requested a telephone interview. The final sample of NLAAS consisted of 4,638 community residing Latino and Asian American adults. The current analyses were limited to the subsample of individuals self-identified as Asians (*N* = 2095).

### Measurement

#### Food Security

Food security was assessed based on three questions regarding access to food. (1) In the past year, how often did you not have enough money to buy food? (Q1), (2) How often in the past 12 months could you not afford to eat balanced meals? (Q2), and (3) How many times in the past 12 months did you cut the size of your meals because there wasn’t enough money to buy food? (Q3). Based on these three questions, we created a binary variable called food security. Those who answered “*never*” to all three questions described above were coded 1 to indicate food secure, while those answered “rarely, sometimes, and often” to Q1 and Q2 were coded 2 to indicate food insecure.

### Depression

The dependent variable of this study was the 12-month Major Depressive Episode (depression hereafter). Respondents who met the depression criteria were coded as “1” for depression and the others “0” for no depression. The presence of depression was identified by the World Health Organization Composite International Diagnostic Interview, a structured interview that follows the criteria of the *Diagnostic and Statistical Manual of Mental Disorders (DSM).* Although the DSM was updated since the survey was conducted, neither the core criterion symptoms applied to the diagnosis of major depressive episode nor the requisite symptom duration of at least 2 weeks has changed (Worley & Matson, 2012).

### Covariates

Three categories of covariates were included. Demographic covariates included age (18–95), gender (man vs. woman), race/ethnicity (Vietnamese, Filipino, Chinese, vs. All other Asian), nativity (US born vs. non-US born), and marital status (married/living with a partner vs. divorced/separated/widowed vs. never married). Socioeconomic covariates included years of education (0–11 vs. 12 vs. 13–15 vs. greater than or equal to 16), poverty status (ratio of family income to the Department of Health and Human Services poverty guidelines greater than one vs. less than one), and health insurance status (have health insurance vs. no health insurance). Health-related covariates included general health status and body mass index (BMI). The general health status was assessed by the question: “*How would you rate your overall physical health?*” Possible responses were excellent, very good, good, fair, or poor. BMIs were calculated by dividing self-reported weight with squared-self-reported height. The results were categorized into six groups, including underweight (BMI less than 18.5), healthy weight (18.5–24.9), overweight (25.0–29.9), obesity class II (35.0–39.9), and obesity class III (greater than 40.0). Lifestyle covariates included smoking, which was quantified by the number of cigarettes smoked per day in the past year (range 0–200).

## Analyses

We used descriptive statistics to estimate population parameters and sample characteristics. To examine the associations between FI and concurrent clinical depression in the past 12 months, we first built a simple logistic regression model with depression as the dependent variable and FI as the independent variable. Then, we sequentially added demographic characteristics, socioeconomic characteristics, and health-related covariates to examine whether the association between FI and depression remained significant after controlling for other characteristics. Finally, to examine potential group variations in the magnitude of the association between FI and depression, we tested the following interaction terms: gender × FI, race/ethnicity × FI, and nativity × FI.

All statistical estimates were weighted, using the recommended NLAAS sampling weight, to ensure the representativeness of the US population. All analyses used the bootstrap resampling technique. Statistical significance was tested at the *α* = 0.05 level, using *F*-tests and adjusted Wald tests.

Those who declined to answer or who answered ‘don’t know’ were counted as missing. All data analyses were performed using the Stata software package version 16. We used publicly available de-identified data. As such, this study was exempt from Institutional Review Board review.

### Population Characteristics

Table 1 describes the sample characteristics. At the time of the study, approximately 34.24% of Asian Americans experienced FI. A greater proportion of self-identified Filipinos and all other Asians experienced FI, compared to their Vietnamese and Chinese counterparts (41.06% vs. 38.53% vs. 26.69% vs. 26.25%), but these differences were not statistically significant. Compared to their once married counterparts, never married Asian Americans experienced greater rates of FI. US-born Asian Americans experienced greater rates of FI compared to their non-US born counterparts (44.17% vs. 31.32%).

**Table 1:** Demographic Characteristics

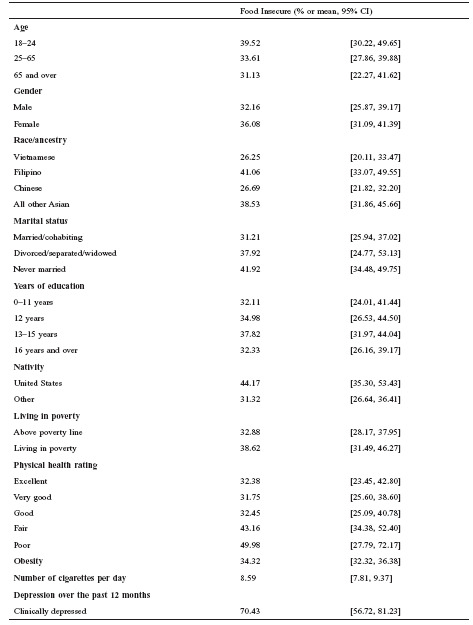

### Association between FI and Depression

Table 2 presents the outcomes of three logistic regression models. We first built model 1 to examine the unadjusted association between FI and depression over the past 12 months. Covariates included in the adjusted models were marital status, nativity, education, living in poverty, having health insurance, and general health status. The odds ratios for having clinical depression over the past 12 months, while experiencing FI in the same period, ranged between 1.62 and 1.44.

**Table 2:** Odds Ratios for Clinical Depression over the Past 12 Months

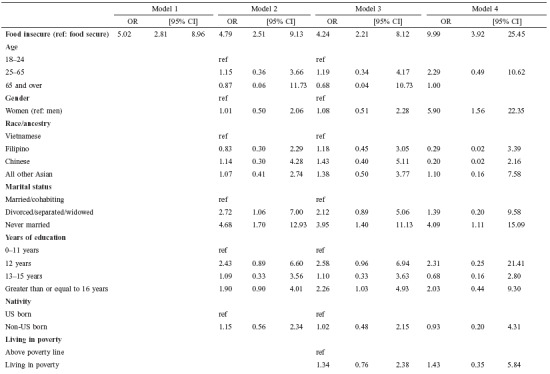	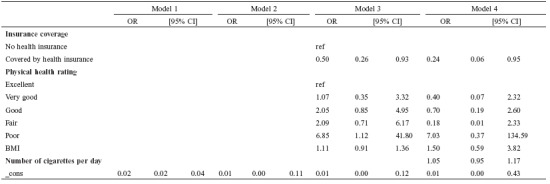

### Differential Magnitudes of the Association between FI and Depression

**Figure 1:**
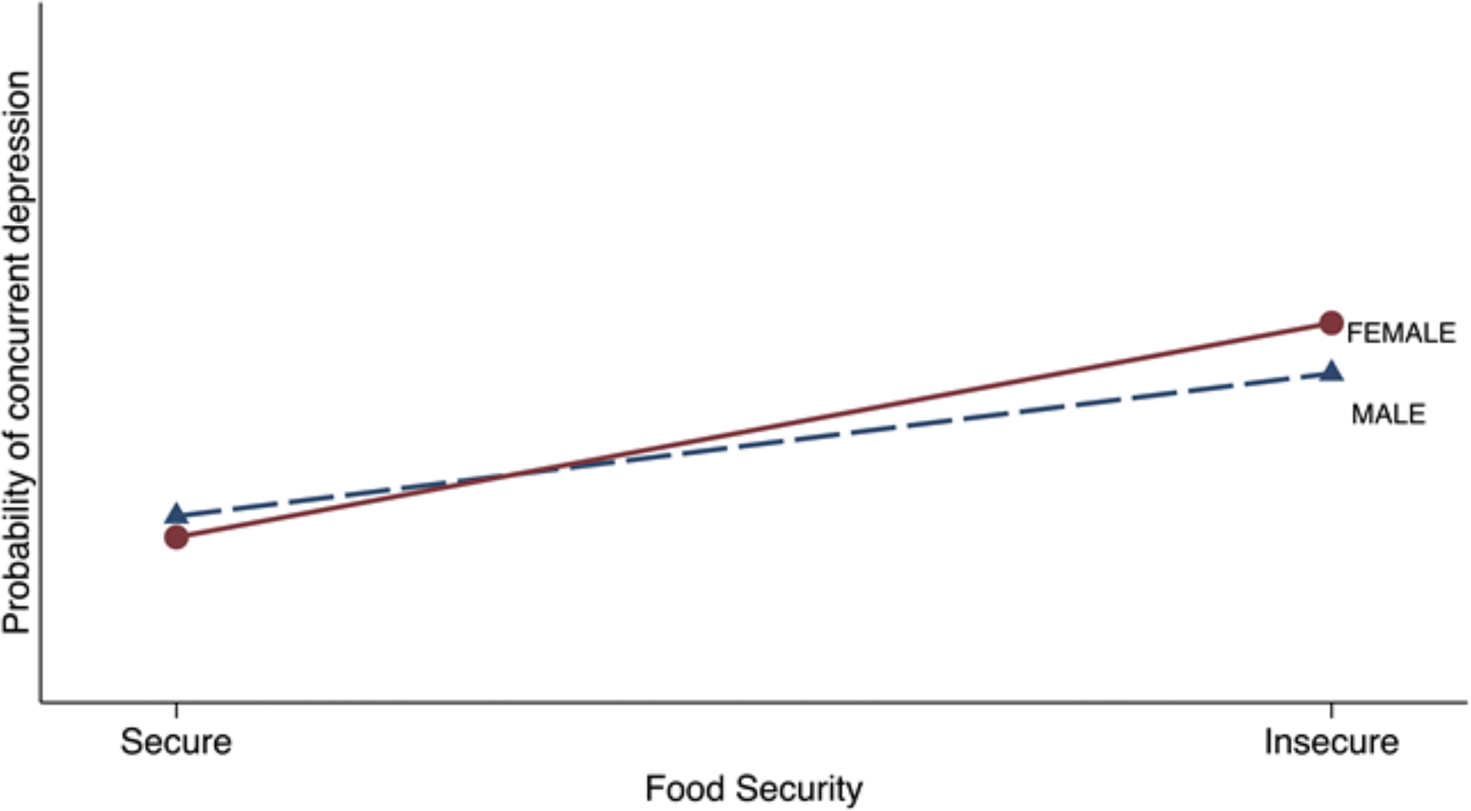
Variations in the associations between food security and depression by gender.

**Figure 2:**
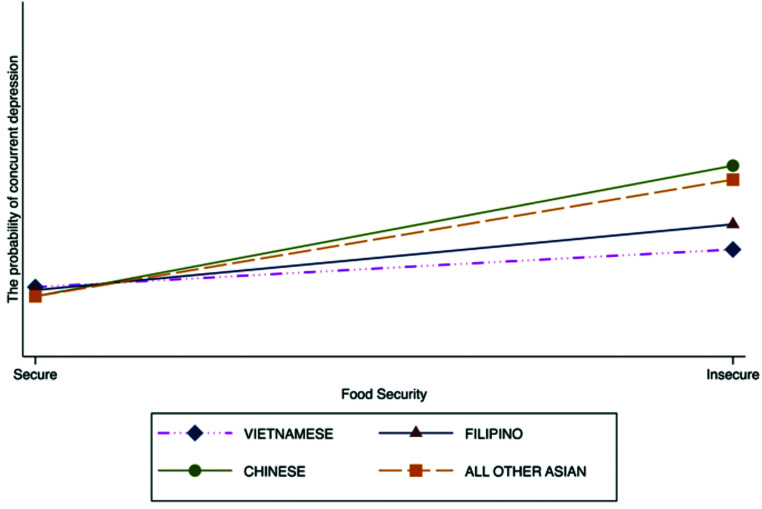
Variations in the associations between food security and depression by race/ethnicity.

**Figure 3:**
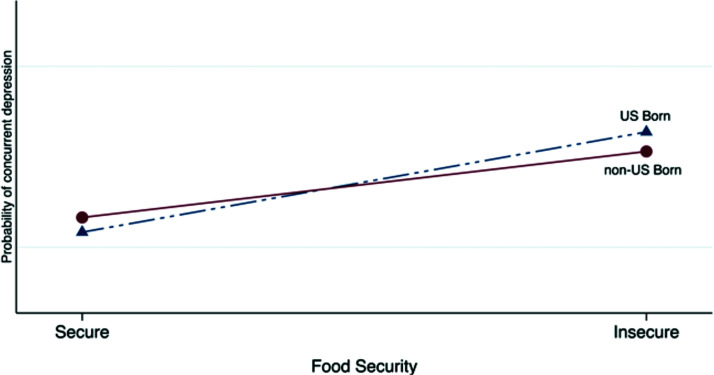
Variations in the associations between food security and depression by nativity.

Table 3 describes varying degrees of adjusted association between FI and depression by race/ethnicity, gender, nativity, and outcomes of adjusted Wald tests for each interaction terms. Figure 1 illustrates that the differential magnitudes of the association between FI and depression was stronger among Asian women than male counterparts (weighted adjusted odds ratio [WAOR]: 6.15 vs. 3.26, *F*(3, 41) = 10.56, *P* < 0.001). Figure 2 indicates that, as expected, the magnitude of the association between FI and depression varies across four Asian American subgroups (Vietnamese, Filipino, Chinese, and All other Asian) (WAOR: 1.82 vs. 2.45 vs. 6.08 vs. 7.61, *F*(7, 47) = 96.53, *P* < 0.01). Finally, Figure 3 shows that among US-born Asian Americans, experiencing FI was associated with a more than 13-fold increase in depression, while among non-US born Asian Americans, the risk was increased by more than 3-fold (WAOR: 13.89 vs. 3.3, *F*(3, 41) = 9.85, *P* < 0.001).

**Table 3:** Variations in the Association Between FI and Depression by Selected Demographic Characteristics

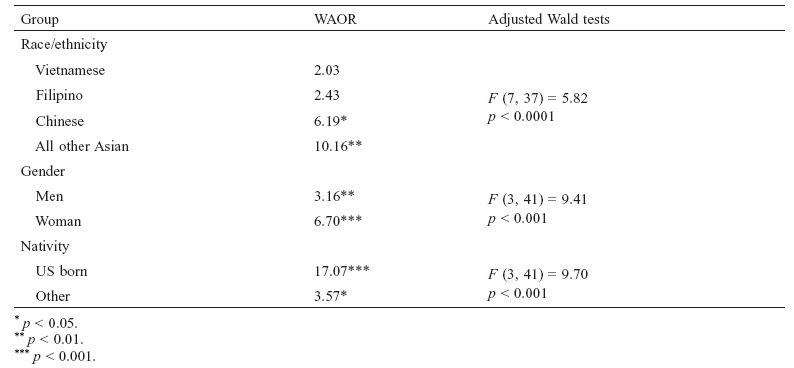

## Discussion

The central findings of this study are that (1) FI significantly increased the risk for clinical depression during the same 12-month period among Asian Americans and (2) magnitudes of said association were greater in certain segments of Asian Americans. The odds of clinical depression were greater among Asian women than Asian men, among US-born than non-US born, and Chinese and other Asians than Filipino and Vietnamese respondents.

The findings of our study are generally congruent with existing literature, in that FI is associated with increased depression and decreased overall mental health in the United States, as well as globally (Arenas et al., 2019; Jones, 2017; Laraia, Vinikoor-Imler, & Siega-Riz, 2015; Lee & Kim, 2019; McLaughlin et al., 2012).

We found that the magnitude of the relation between FI and depression is greater in Asian women than in Asian men. Few medical and epidemiological studies have assessed potential mechanisms to explain the gender variations observed in this study. However, considerable data confirm gender differences in mental health, health-seeking behaviors, health outcomes, and healthcare utilization (Becerra et al., 2018). Several reasons can be contributed to the gender variations in the magnitude of the association between FI and depression observed in this study. First, FI can lead to poor diet and nutritional deficiencies, which then trigger biological responses that lead to depression. Second, FI may increase feelings of hopelessness and a sense of powerlessness, which can contribute to the elevation of anxiety and depression. Finally, gender roles are known to impact gender differently and in diverse ways (Mayor, 2015). Regarding traditional gender roles in Asian culture, women are expected to care for household members’ health and wellbeing. Food insecurity within an Asian household may increase depressed feelings for women, as optimizing nutritional status is one of their responsibilities.

We observed variations in rates of FI and of depression, and the association between FI and depression across different subgroups of Asian Americans. First, while Filipino Americans experienced the highest rates of FI among the Asian American sub-groups, the magnitude of the association between FI and depression was strongest among Chinese Americans and all other Asian groups. Existing literature does not provide sufficient information to explain why FI impacts certain Asian groups more than others in terms of depression. Potential explanations may include cultural meaning of food and food insecurity, and dietary habits. However, more research is needed to discern the potential mechanism of the differential association between FI and depression.

Also, differential rates of FI across different Asian groups were observed in California (Becerra et al., 2018). However, it is unclear how and why FI impacts different Asian groups variously. Further studies are needed to replicate our results and identify underlying mechanisms for why experiencing FI is associated with poor mental health outcomes more strongly among certain ethnic groups than others.

US-born Asian Americans experienced a greater magnitude of the association between FI and depression than non-US born Asian Americans. Several reasons could contribute to this finding. For US-born Asian Americans, food security may be part of an expectation to be successful and productive member of society. Therefore, FI could be perceived as a failure to meet societal expectations and may increase the likelihood of depression. On the other hand, immigrants might be more likely than US-born Asian Americans to normalize FI. It may be perceived as a conventional hardship that occurs with the acculturation process, thus lowering the likelihood of depression. Another possibility could be that while US-born Asian Americans may be more acculturated and therefore more individualistic, their non-US born counterparts may retain more communal lifestyles due to collectivist cultural identities. Therefore, the varying levels of acculturation may impact how one receives social and familial support when experiencing FI. Those who have communal lifestyles may have stronger support systems that protect them from depression.

### Limitation and Strength of the Study

Due to the cross-sectional nature of this study, the causal inference should not be drawn from this study. Also, the design of the NLAAS survey did not allow us to examine all other Asian groups, which may include highly vulnerable populations. Furthermore, the rate of FI and depression may have been underestimated due to underreporting of food-insecure status. The findings of this study should be viewed in the historical context because data of NLAAS was collected between May 2002 and November 2003. Several societal changes since the data collection may have also impacted how FI and clinical depression are associated. Finally, detailed information about diet and neighborhood environment, both of which affect FI and depression, were not included in NLAAS (Chung et al., 2012; Montgomery et al., 2017).

### Strength and Contribution of the Study

Despite the above limitations, this study has several strengths. First, this study used a nationally representative sample of older adults from major Asian groups. Second, this study used the clinical diagnosis of depression—increasing the clinical relevancy of our findings. Finally, the current study adds to the knowledge base regarding FI and depression in diverse Asian populations in the United States. As previously mentioned, few existing studies have examined how FI impacted diverse Asian American subgroups. The current study contributes to filling the aforementioned gap in the literature. To our knowledge, this is the first study to assess the differential associations between FI and depression by gender and race/ethnicity of a representative sample of the US Asian population.

## Conclusions

Greater attention is needed towards food-insecure Asian Americans and their mental health. While FI impacts all Asian Americans, certain segments of Asian population may be impacted more than others. Thus, providers who care for Asian Americans might need to place a higher priority on FI screening in order to help their patients maintain optimal nutritional and mental health.

## Acknowledgments

A previous version of this study was presented at the 21st International Association of Gerontology and Geriatrics. At the time of the study, Dr. Park was supported by National Institute of Health (K01NR015101).

## Declaration of Conflicting Interests

The authors declared no potential conflicts of interest concerning the research, authorship, or publication of this article.

## Funding

This study was supported by the National Institute of Health (K01NR015101).
